# Effects of Different Particle Sizes of Ultrafine Indica Rice Flour and *Lactobacillus casei* on the Fermentation and Flavor Characteristics of Danling Dongba

**DOI:** 10.1002/fsn3.71113

**Published:** 2025-10-24

**Authors:** Hong Jiang, Chong Liu, Hailun Wan, Hongyu Li, Zhangkui Yan, Xiaohong Wen, Wanqing Feng, Can Tang, Yong Tang

**Affiliations:** ^1^ College of Food and Bioengineering Xihua University Chengdu China; ^2^ Sichuan Kelu Pnarmaceutical Co. Ltd. Chengdu China; ^3^ Chengdu Second Peoples' Hospital Chengdu China; ^4^ Sichuan Yaxiangju Food Technology Co. Ltd Meishan China

**Keywords:** Danling Dongba, *Lactobacillus*, superfine indica rice flour, volatile flavor

## Abstract

This study aimed to enhance the flavor and quality of Danling Dongba, a traditional fermented rice product, by preparing ultrafine indica rice flours using fluidized bed airflow crushing. Flours with particle sizes of 20.16, 17.16, and 12.22 μm were fermented with *Lactobacillus plantarum* 550 ( *L. plantarum* 550), *Lactobacillus fermentum GF1800* ( *L. fermentum* GF1800), and *Lactobacillus paracasei S6 (L. paracasei* S6) under optimized conditions. The 12.22 μm flour showed the best performance, with a water absorption index of 2.32 g/g, solubility of 2.05%, swelling power of 9.26 g/g, and peak viscosity of 7632.53 cP. Solid‐phase microextraction coupled with GC × GC–MS revealed that *Lactobacillus* fermentation increased volatile flavor compounds from 42 (unfermented) to 72. Notably, *L. plantarum* 550 enhanced fruity esters such as ethyl caprylate (1.18%), while *L. paracasei* S6 enriched milk‐like ketones such as 3‐hydroxy‐2‐butanone (1.54%). These findings demonstrate that optimizing flour fineness and *Lactobacillus* strains can significantly improve the flavor complexity of Danling Dongba, offering a scientific basis for its standardized production and industrial development.

## Graphical abstract

This study found that ultrafine indica rice flour (12.22 μm) exhibited superior hydration properties (WAI 2.32 g/g, WSI 2.05%, SP 9.26 g/g) and higher pasting viscosity (peak viscosity 7632.53 cP). *Lactobacillus* fermentation increased volatile flavor compounds from 42 to 72, with *L. plantarum* 550 enhancing fruity esters and *L. paracasei* S6 enriching creamy ketones. These findings provide a scientific basis for optimizing the flavor and standardizing the production of Danling Dongba.

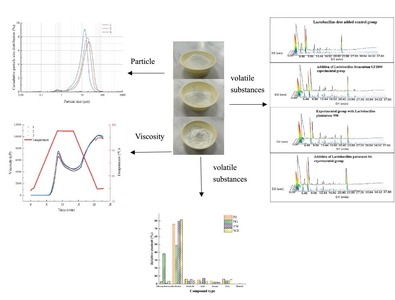

## 1 Introduction

Danling Dongba, a traditional indica rice‐fermented cake originating from Danling, Sichuan, China, is prized for its unique flavor and textural properties. As a naturally gluten‐free food, it holds significant value in meeting dietary requirements of gluten‐intolerant populations, particularly those with celiac disease (Hosseini et al. 2018). However, traditional production relies on empirical operations and an unstable microbial consortium in the “old slurry” starter culture, resulting in substantial product variability and elevated food safety risks (e.g., mold contamination and over‐fermentation) (Yee et al. 2025). Additionally, its characteristically high sugar and fat content conflicts with contemporary health trends, while a short shelf‐life restricts market distribution (Jahn et al. 2023).

Building upon pre‐optimized fermentation parameters, this study established a stable and reproducible fermentation system. Within this controlled framework, implementing direct‐vat‐set (DVS) starter cultures constitutes the core strategy for enhancing product quality and safety (Wang et al. 2025). Research demonstrates that synergistic interactions between yeast and specific lactic acid bacteria ( *L. plantarum* , *L. fermentum* , and *L. paracasei* ) reduce fermentation time by ~50% while improving textural properties (Adesulu‐Dahunsi et al. 2020). This microbial application paradigm has been widely adopted in industrial‐scale production of fermented foods such as yogurt and bread (Jung et al. 2024).

Despite the technological maturity of DVS starters and process stability, the impact of specific microbial combinations on volatile flavor profiles—a core quality attribute of Danling Dongba—remains unclear. Current research predominantly focuses on process optimization or raw material modification (Shu et al. 2025), lacking systematic analysis of strain‐specific metabolic contributions to flavor development.

Therefore, this study aimed to investigate the effects of particle size of superfine indica rice flour on the physicochemical properties of indica rice slurry, first to identify the optimal size range for industrial production. Then, using our previously established standardized fermentation system, the influence of a defined yeast–LAB co‐culture (direct‐vat starter) on the volatile profile of Danling Dongba fermented indica rice slurry was elucidated by solid‐phase microextraction coupled with comprehensive two‐dimensional gas chromatography–mass spectrometry (SPME‐GC×GC–MS).

## 2 Materials and Methods

### 2.1 Preparation of Ultrafine Indica Rice Flour

The fine particles were collected via the airflow mill's cyclone separator, where centrifugal classification effectively segregated particles by size. Larger particles were recirculated to the grinding chamber for further comminution, while fines were directed to the collection chamber. Initial milling yielded particles averaging 30–50 μm. To achieve sub‐30 μm particles, we optimized three key parameters: nozzle stagnation pressure, nozzle‐to‐plate gap distance, and feed rate. This protocol successfully generated three distinct grades of ultrafine indica rice flour with controlled particle size distributions.

### 2.2 Determination of Particle Size by Laser Particle Sizer

The particle size distribution of the ultrafine indica rice flour samples was determined using a laser diffraction particle size analyzer (Bettersize2600, Dandong, Shandong, China) (Sun et al. 2023). Three groups of ultrafine indica rice flour samples with different particle sizes were analyzed using a dry feeding method. To verify instrument accuracy, a standard corundum (Al_2_O_3_) reference sample was used for calibration. Measurements were performed in triplicate, yielding *D*_10_, *D*_50_, and *D*_90_ values of 69.46 ± 0.025 μm, 107.5 ± 0.019 μm, and 161.3 ± 0.023 μm, respectively. These values fell within the certified range of the standard, confirming the instrument's reliability under the specified conditions. Following this confirmation, the instrument autonomously selected an appropriate test mode to analyze the rice flour samples, ensuring precise and consistent measurements.

### 2.3 Water Absorption Index, Water Solubility Index, Swelling Power and Oil Absorption Capacity

The Water Absorption Index (WAI), Water Solubility Index (WSI), Swelling Power (SP), and Oil Absorption Capacity (OAC) were quantified according to the method of Meng and Kim (2020) with minor modifications. For WAI and WSI, ultrafine indica rice flour (1.00 ± 0.01 g) was dispersed in 12 mL of distilled water (1:12, w/v) and incubated in a shaking water bath at 30°C for 30 min (agitation every 5 min). The slurry was then centrifuged at 3000 rpm for 15 min. The supernatant was collected, dried to constant mass at 105°C, and weighed. WAI was calculated as the mass of the hydrated sediment per gram of dry flour, whereas WSI was expressed as the percentage of dry solids recovered in the supernatant relative to the initial sample mass.

SP was determined by mixing 1.0 g of rice flour with 40 mL of distilled water, heating the mixture in a water bath at 85°C for 30 min, and then centrifuging it at 3000 × g for 15 min. After cooling to room temperature, SP was calculated as the ratio of the wet gel mass to the original sample mass.

For OAC determination, rice flour was mixed with corn oil at a ratio of 1:10 (w/v), vortexed for 1 min, and equilibrated at room temperature for 30 min. The mixture was then centrifuged at 4000 rpm for 25 min. After carefully decanting the oil layer, the centrifuge tube was placed at a 45° angle for 1 h and subsequently reweighed. OAC was expressed as the percentage of oil absorbed per 100 g of the sample. The formulas used for calculation are provided below:

(1) $$X_{a}=\frac{m_{1}-M}{M}$$

(2) $$X_{b}\left(\%\right)=\frac{m_{2}}{M}\times 100$$

(3) $$X_{c}=\frac{m_{1}}{M}$$

(4) $$X_{d}=\frac{m_{i}-m_{j}}{m_{j}}\times 100$$

Note: *X*_*a*_ is water absorption index (WAI), g/g; *X*_*b*_ is water solubility index (WSI), %; *X*_*c*_ is swelling power (SP), g/g; *X*_*d*_ is oil absorbing capacity, %. *m*_1_ is the weight of precipitation, g; *m*_2_ is the dry weight of supernatant, g; *M* is the dry weight of the sample, g; m_i_ is the weight of the lower layer of precipitation, g; *m*_*j*_ is the dry weight of the sample, g.

### 2.4 Determination of Rheological Properties

The static rheological properties of ultrafine indica rice flour were assessed using a method modified from Oh et al. (2024). Three distinct particle size groups of the flour were dispersed in distilled water at a 3:25 (w/v) ratio using a vortex mixer. Rheological characterization was performed using a stress‐controlled rheometer (Anton Paar MCR 301). The temperature profile consisted of: an initial equilibration at 50°C for 50 s; heating to 95°C at 6.0°C/min; a 5 min hold at 95°C; cooling back to 50°C at 6.0°C/min; and a final hold at 50°C for 2 min. Dynamic temperature rotational scanning was conducted at a constant rotational speed of 100 min^−1^ (approximately 100 rpm). A starch‐specific concentric cylinder geometry (ST24‐2D/2 V) was employed, with a rotor diameter of 24 mm, rotor length of 30 mm, and a zero gap setting. Key rheological parameters measured included pasting temperature, peak viscosity, breakdown viscosity, setback viscosity, and final viscosity.

Dynamic rheological properties were determined following the method described by von Borries‐Medrano et al. (2019). Samples were prepared by dispersing the material in distilled water at a ratio of 1:3 (w/v). Amplitude sweeps were initially performed to identify the linear viscoelastic region (determined at 0.1% strain). Subsequently, temperature sweeps were conducted in oscillatory mode at a heating rate of 2.0°C/min up to 95°C, followed by an 8 min isothermal hold at this temperature. Measurements employed a parallel plate geometry (PP50 rotor, 50 mm diameter) with a 1.0 mm gap; samples were sealed with silicone oil to prevent evaporation. Storage modulus (G′), loss modulus (G″), and loss tangent (tan δ) were recorded at a constant strain of 0.1% and an angular frequency of 10 rad/s. All measurements were performed in triplicate.

### 2.5 Measurement of Thermal Properties

The thermal properties of three groups of ultrafine indica rice flours with different particle sizes were analyzed using a Mettler differential scanning calorimeter (DSC, Mettler Toledo DSC 3) following the methodology described by Wang et al. (2022), accurately weighing 6.0 mg of ultrafine indica rice flour (precision: ±0.1 mg), mixed with distilled water at a 1:3 (m/v) ratio, and hermetically sealed in an aluminum crucible. Equilibrate the mixture at room temperature for 24 h. Thermal analysis was performed from 25°C to 100°C at a constant heating rate of 10°C/min, using an empty aluminum crucible as a reference. Triplicate measurements were conducted for each sample group.

### 2.6 Indicaindica Rice Slurry Fermentation Conditions and Strain Inoculation

To standardize fermentation prior to volatile compound analysis, indica rice slurry was prepared using No. 3 superfine indica rice flour (D_50_ = 12.22 μm), mixed with distilled water (1:1.42, w/v), and supplemented with 30% (w/w) sucrose and 5% (w/w) soybean milk flour. The slurry was pre‐sterilized (100°C, 30 min), and all procedures were conducted under aseptic conditions using sterile equipment. Fermentation was performed at 24°C with 0.45% (w/w) active dry yeast and 0.05% (v/w) of either *L. plantarum* 550, *L. fermentum* GF1800, or *L. paracasei* S6 (1.0 × 10^11^ CFU/g), and was terminated upon reaching pH 5.37. A yeast‐only control group was included under identical conditions (24°C, 75% relative humidity) to ensure consistency across treatments. Fermentation progress was monitored by regular pH measurements, and no visible signs of contamination, such as abnormal odors or discoloration, were observed throughout the process.

### 2.7 Solid Phase Microextraction Conditions

Volatile compounds in four groups of fermented indica rice slurry were analyzed using a method modified from Zhao et al. (2023). The groups included samples inoculated with *L. plantarum* 550, *L. fermentum* GF1800, *L. paracasei* S6, and a non‐inoculated control group. Solid‐phase microextraction (SPME) coupled with comprehensive two‐dimensional gas chromatography–mass spectrometry (GC×GC/MS; 7820A/5977E MSD, Agilent Technologies, Santa Clara, CA, USA) was utilized for analysis.

Briefly, 1 g of fully fermented indica rice slurry was weighed into a 15 mL headspace vial, sealed, and equilibrated in a water bath at 55°C. Volatiles were extracted using a 65 μm polydimethylsiloxane/divinylbenzene (PDMS/DVB) fiber for 30 min, followed by thermal desorption in the GC injection port at 250°C for 3 min.

Chromatographic separation was achieved on an Agilent HP‐5 quartz capillary column (30 m × 0.32 mm, 0.25 μm) with high‐purity helium as the carrier gas at a constant flow rate of 1.00 mL/min under splitless injection mode. The inlet temperature was maintained at 250°C. The column temperature program was as follows: initial hold at 40°C for 1 min; ramp to 120°C at 6°C/min with a 5 min hold; then ramp to 250°C at 10°C/min with an 8 min hold.

Mass spectrometric detection was performed in electron ionization (EI) mode with an electron energy of 70 eV. The ion source and interface temperatures were set to 230°C and 250°C, respectively, and the mass scan range was 30–450 m/z. Identification of GC×GC–MS peaks was conducted by comparing mass spectra with those in the NIST 17 database. All experiments were performed in quadruplicate to ensure reproducibility.

### 2.8 Statistical Analysis

Duncan's multiple range test was performed using SPSS 26.0 software at a significance level of 0.05 to determine significant differences among group means. Descriptive data processing was conducted using Excel 2016. All statistical results are expressed as mean ± standard deviation based on six independent measurements.

## 3 Results and Discussion

### 3.1 Particle Size Distribution of Ultrafine Indica Rice Flour

As shown in Table 1, airflow milling exerted a significant effect on the particle sizes of the three groups of ultrafine indica rice flour samples. The D90 values revealed that 90% of the particles had a size below 35 μm, with an average particle size ranging from 11 to 20 μm. Among the samples, sample No. 3 exhibited the smallest particle sizes, with *D*_[4,3]_, *D*_[3,2]_, *D*_50_, and *D*_90_ being 12.61 ± 0.110 μm, 7.18 ± 0.021 μm, 12.22 ± 0.098 μm, and 20.86 ± 0.214 μm, respectively. In contrast, sample No. 1 had the largest particle size, with a *D*_50_ of 18.75 ± 0.181 μm, and the difference between its *D*_[4,3]_ and *D*_50_ values was 1.41 μm. These results indicate that airflow milling effectively reduced the particle size of indica rice flour and yielded particles with a uniform distribution (Zhang et al. 2024).

**TABLE 1:** Ultrafine indica rice flour particle size results.

Sample number	*D*_[4,3]_ (μm)	*D*_[3,2]_ (μm)	*D*_50_ (μm)	*D*_90_ (μm)
1	20.16 ± 0.180^a^	10.2 ± 0.193^a^	18.75 ± 0.181^a^	34.96 ± 0.163^a^
2	17.16 ± 0.025^b^	9.09 ± 0.061^b^	15.98 ± 0.006^b^	28.83 ± 0.159^b^
3	12.61 ± 0.110^c^	7.18 ± 0.021^c^	12.22 ± 0.098^c^	20.86 ± 0.214^c^

*Note:* Volume average diameter *D*_[4,3]_ indicates the weighted average of particle size to particle weight (volume), area average diameter *D*_[3,2]_ indicates the weighted average of particle size to particle surface area, and *D*_50_ and *D*_90_ indicate that on the cumulative distribution curve of particle size, 50% and 90% of the particle diameter are smaller than this value. Different superscript letters (a, b, c) within a column indicate statistically significant differences between the sample means (p < 0.05).

**FIGURE 1:**
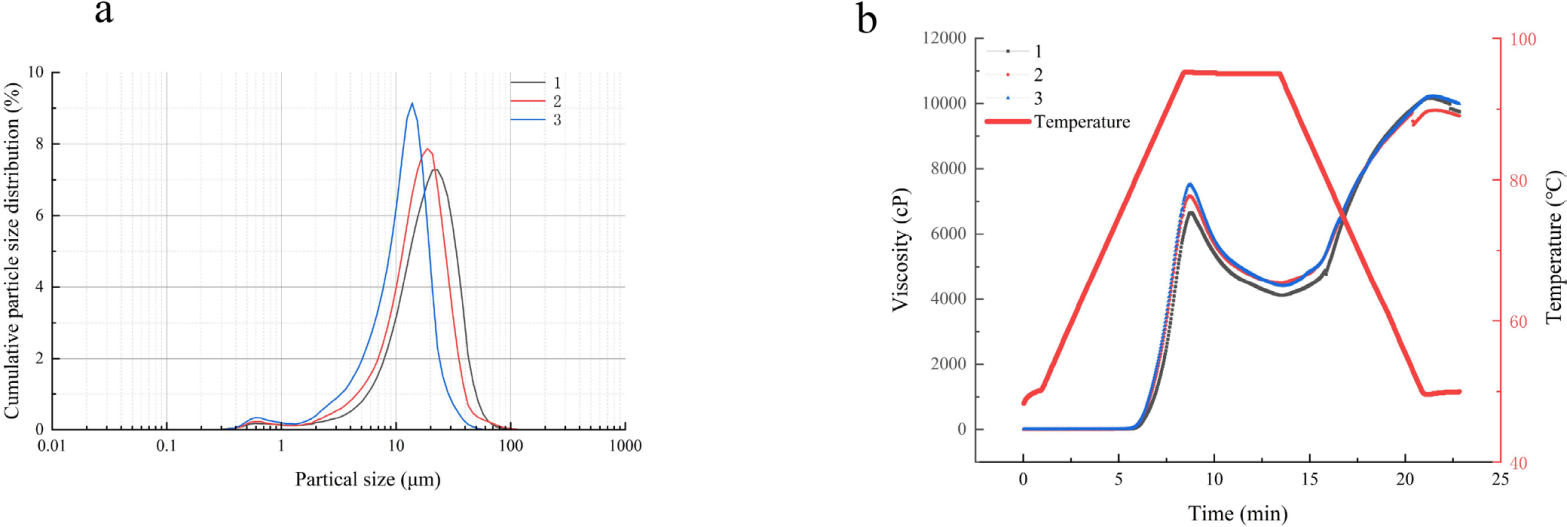
The particle sizes distribution of the superfine Indica rice powders (a). Viscosity changes of 3 groups of superfine indica rice flour samples with different particle sizes during gelatinization (b).

As particle size decreases, the specific surface area of particles increases, thereby enhancing interparticle contact. This augmentation in contact strengthens interparticle interactions—including van der Waals forces, electrostatic forces, and liquid bridge forces—which are more prominent in smaller particles. Such strengthened interactions elevate flow resistance, resulting in a higher viscosity of the slurry (Zheng et al. 2024). Additionally, a narrower particle size distribution (as observed in sample No. 3) (Figure 1a) facilitates more efficient particle packing, reducing the free space between particles and further contributing to the increased viscosity (Caputo et al. 2019). Such improved particle uniformity is expected to influence the rheological behavior of the system, which is further examined in Section 3.3.

### 3.2 Analysis of the Hydration Properties of Ultrafine Indica Rice Flour

As shown in Table 2, no significant difference was observed in the swelling power (SP) among the three groups of ultrafine indica rice flour samples with varying particle sizes. This indicates that for samples with an average particle size below 20 μm, the differences in volume expansion after gelatinization were minimal under conditions of sufficient moisture availability.

**TABLE 2:** Results of hydration characterization of ultrafine indica rice flour.

Sample number	WAI (g/g)	WSI (%)	SP (g/g)	Oil absorption capacity (%)
1	2.24 ± 0.01^b^	1.68 ± 0.04^c^	8.74 ± 0.03^a^	82.53 ± 1.26^c^
2	2.28 ± 0.01^ab^	1.90 ± 0.08^b^	8.91 ± 0.01^a^	87.17 ± 0.93^b^
3	2.32 ± 0.04^a^	2.05 ± 0.06^a^	9.26 ± 0.57^a^	90.50 ± 0.95^a^

*Note:* ^a,b^Means in the same column followed by different letters are significantly different (*p* < 0.05) according to Duncan's multiple range test.

Furthermore, the pulverization process improved the solubility of indica rice flour. Both the water absorption index (WAI) and solubility increased with decreasing particle size. This enhancement is likely attributed to the smaller particle size and higher particle uniformity (Lapcíková et al. 2021). The water solubility index (WSI) and oil absorption capacity (OAC) exhibited a similar trend, with both parameters increasing significantly as particle size decreased. This phenomenon may be explained by the exposure of more polar or charged side chains, which enhances the solubility of starch granules. Additionally, it is known that the hydrophilic moieties of proteins and carbohydrates contribute to water absorption capacity, whereas the hydrophobic regions of proteins interact with lipids to promote oil absorption capacity. These findings suggest that ultrafine pulverization facilitates the release of particulate proteins, thereby modulating both water and oil absorption properties (Hu et al. 2024).

### 3.3 Pasting Characterization of Ultrafine Indica Rice Flour

The primary component of super indica rice flour is starch, which comprises amylose (linear starch) and amylopectin (branched starch). Prior to gelatinization, starch granules remain suspended in water (Zhang et al. 2021). Figure 1b depicts the viscosity changes of the three sample groups across different temperatures. At lower temperatures, starch granules retained their native structure. As temperature increased, the granules absorbed water, swelled, and their volume fraction increased, causing a rapid rise in viscosity until a peak was reached. The peak viscosity followed the order: No. 3 > No. 2 > No. 1. This indicates that viscosity increased with decreasing particle size, likely due to strengthened interparticle interactions. At a constant temperature, viscosity decreased as a result of starch granule disruption and reduced volume fraction. During cooling, viscosity increased again because the rupture of granules released amylose (linear starch), which promoted the gelatinization of the starch dispersion system and thus led to a viscosity increase (Islam et al. 2024).

Five key pasting parameters—peak viscosity (PV), pasting temperature (PT), breakdown viscosity (BV), final viscosity (FV), and setback viscosity (SV)—were extracted from the flow curves (Table 3). PV rose markedly (*p* < 0.05) with decreasing particle size, reaching a maximum of 7633 ± 98 cP in sample No. 3. The pronounced increase can be ascribed to the larger specific surface area that facilitates water imbibition and starch solubilization (Lee et al. 2021). PT remained statistically invariant across all size fractions, indicating that granule swelling onset is independent of particle size under the employed heating rate. Conversely, BV—a proxy for paste thermal stability—also escalated as particle size diminished, implying that excessive comminution compromises structural robustness at elevated temperatures. Upon cooling, FV, which reflects starch re‐association, did not differ significantly among the three samples, whereas SV, an index of retrogradation tendency, increased sharply (*p* < 0.05) with finer particles. This behavior is consistent with enhanced leaching of linear amylose chains that readily re‐crystallize during cooling.

**TABLE 3:** Characteristic parameters of pasting of three groups of ultrafine indica rice flour samples with different grain sizes.

Sample number	PT (°C)	cP			
		PV	BV	FV	SV
1	77.63 ± 0.06^a^	6484.33 ± 201.09^c^	2398.67 ± 90.78^c^	9687.33 ± 119.0^a^	2350.67 ± 92.68^c^
2	77.33 ± 0.12^a^	7006.33 ± 143.27^b^	2585.67 ± 64.38^b^	9705.67 ± 71.70^a^	2714.0 ± 143.43^b^
3	77.07 ± 0.06^a^	7632.53 ± 98.47^a^	3164.67 ± 39.58^a^	9709.67 ± 71.28^a^	3217.67 ± 96.96^a^

*Note:* ^a,b^Means in the same column followed by different letters are significantly different (*p* < 0.05) according to Duncan's multiple range test.

### 3.4 Dynamic Rheological Characterization of Ultrafine Indica Rice Flour

**FIGURE 2:**
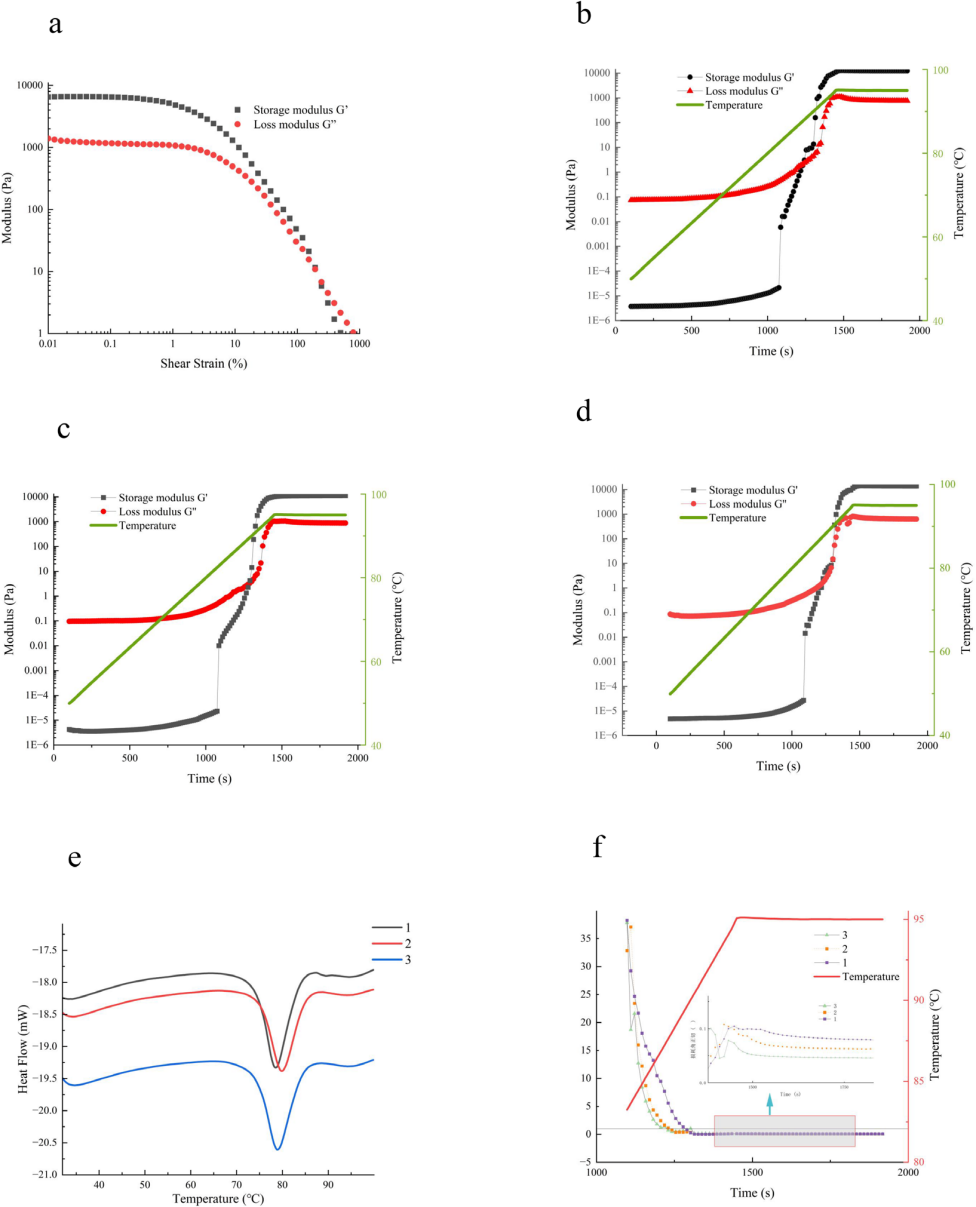
Changes of storage energy and loss modulus of superfine indica rice flour when the logarithm of strain changes (a–d are the dynamic viscoelasticity diagrams of No. 1, No. 2, and No. 3 supermicro indica rice samples, respectively). The tan δ diagrams of three groups of supermicro indica rice samples with different grain sizes at 83°C–95°C (e). Thermal characteristics of 3 groups of superfine indica rice flour with different particle sizes from 25°C to 100°C (f).

Figure 2a presents the amplitude sweep results for ultrafine *indica* rice flour, depicting the relationship between strain (0.01–1000%, logarithmic scale) at a fixed angular frequency (ω = 10 rad/s). The storage modulus (G′) represents the material's elastic component, quantifying its ability to recover energy from deformation; higher G′ values indicate greater elastic solid‐like behavior. Conversely, the loss modulus (G″) represents the viscous component, characterizing energy dissipation and resistance to flow; larger G″ values signify stronger viscous liquid‐like behavior (Xiong et al. 2021).

As shown in Figure 2a, the linear viscoelastic region (LVR) for the ultrafine *indica* rice flour extends up to a strain of approximately 1%. Within the LVR, the material's internal structure remains intact, stress is proportional to strain, and both moduli (G′ and G″) remain constant (Jiao et al. 2020). Throughout this LVR, G′>G″ for the sample, indicating dominant elastic behavior, gel‐like characteristics, and classification as a viscoelastic solid.

As strain increases beyond the LVR (> 1%), structural breakdown occurs. Initially isolated microcracks propagate and eventually coalesce, forming a continuous macroscopic failure plane across the sample or the measurement system's shear gap. This network collapse results in the dominance of viscous flow. Beyond the strain where G′ = G″ (the crossover point), G″>G′, signifying a transition to fluid‐like behavior where viscous dissipation predominates (Jastram et al. 2021).

Figure 2b–e depict the dynamic rheological evolution of three ultrafine *indica* rice flour groups with distinct particle sizes during heating and isothermal stages. As shown in Figure 2b–d, both storage (G′) and loss (G″) moduli increased progressively with temperature for all samples before stabilizing during the 95°C isothermal hold. This modulus enhancement during heating is attributed to particle swelling and the consequent increase in starch volume fraction, which initiates gel network formation upon reaching critical temperatures.

The sol–gel transition temperatures—identified by the G′/G″ crossover—occurred at 86.82°C (Sample A), 87.22°C (Sample B), and 88.43°C (Sample C), corresponding to transition times of 18.0, 18.6, and 19.2 min, respectively, from the heating onset. These results demonstrate that particle size variations minimally influence the fundamental sol‐to‐gel transformation pathway, with all samples exhibiting comparable gelation kinetics despite minor temporal differences.

As shown in Figure 2e, the loss tangent (tan δ) decreased toward unity during heating, indicating the crossover where G′<G″. This transition marks the onset of internal gelation, characterized by rapid morphological transformation from liquid to gel state (Wu et al. 2023). Throughout the isothermal phase at 95°C, tan δ remained stable below 1. Notably, tan δ values exhibited a slight positive correlation with decreasing particle size, ranging from 0.05 to 0.10 across samples. According to Arlai and Tananuwong (2021), tan δ serves as a sensitive indicator of viscoelastic changes in polymer gels, with values < 0.1 typically signifying well‐crosslinked networks. These results collectively demonstrate that all ultrafine *indica* flour gels developed robust reticular structures. However, Sample 3 exhibited marginally reduced rigidity (higher tan δ ≈0.10), suggesting a less densely crosslinked network. This structural distinction aligns with Sample 3's technological performance: Its superior Peak Viscosity (PV) and Final Viscosity (FV) values indicate enhanced potential for rehydrated fermentation—a critical attribute for fermented *indica* rice products like Danling Dongba, where optimal bubble structure preservation during cooking determines the desired fluffy, glutinous texture.

### 3.5 Thermal Characterization of Ultrafine Indica Rice Flour

Table 4 summarizes the thermal transition parameters of ultrafine *indica* rice flours with varying particle sizes, including onset temperature (To), peak temperature (Tp), conclusion temperature (Tc), gelatinization enthalpy (ΔH), and pasting range (ΔT). A consistent inverse relationship emerged between particle size and pasting temperatures: To, Tc, and ΔT decreased progressively with reduced particle dimensions, reaching minima of 73.89°C ± 0.04°C, 82.83°C ± 0.05°C, and 8.95°C ± 0.09°C, respectively. This reduction stems from higher starch granule density per unit volume in finer particles, which enhances water accessibility and lowers the thermal energy required for gelatinization (Niu et al. 2019).

**TABLE 4:** Thermal characterization parameters of 3 groups of ultrafine indica flour with different grain sizes.

Sample	Thermal parameter/°C				
	*T*_*o*_	*T*_*p*_	*T*_*c*_	ΔH (J/g)	ΔT
1	74.74 ± 0.04^a^	78.16 ± 0.08^b^	83.87 ± 0.07^a^	1.70 ± 0.03^a^	9.13 ± 0.11^b^
2	73.95 ± 0.07^b^	79.23 ± 0.05^a^	83.38 ± 0.04^b^	1.42 ± 0.05^b^	9.43 ± 0.06^a^
3	73.89 ± 0.04^b^	79.20 ± 0.06^a^	82.83 ± 0.05^c^	1.22 ± 0.05^c^	8.95 ± 0.09^c^

*Note:* Different letters indicate significant differences in means within columns. ΔT = *T*_*c*_−*T*_*o*_.

Furthermore, all three samples exhibited reduced peak temperatures (Tp), conclusion temperatures (Tc), and pasting ranges (ΔT). This trend aligns with reported reductions in pasting temperatures for dry‐milled rice starch systems, where elevated damaged starch content enhances water absorption capacity and facilitates gelatinization at lower temperatures (Gao et al. 2024). The gelatinization enthalpy (ΔH) decreased significantly from 1.70 ± 0.03 J/g (Sample 1) to 1.22 ± 0.05 J/g (Sample 3), confirming diminished energy requirements for pasting with decreasing particle size. This reduction likely originates from progressive mechanical damage during size reduction, which compromises the structural integrity of remaining crystalline lamellae in starch granules.

The DSC thermograms in Figure 2f exhibit a single endothermic peak for all samples, corresponding to starch gelatinization under excess water conditions. These results demonstrate that particle size reduction in ultrafine *indica* flour substantially modifies its gelatinization characteristics.

### 3.6 Total Ion Chromatograms of Different *Lactobacillus*‐Fermented Indica Rice Slurry Samples

**FIGURE 3:**
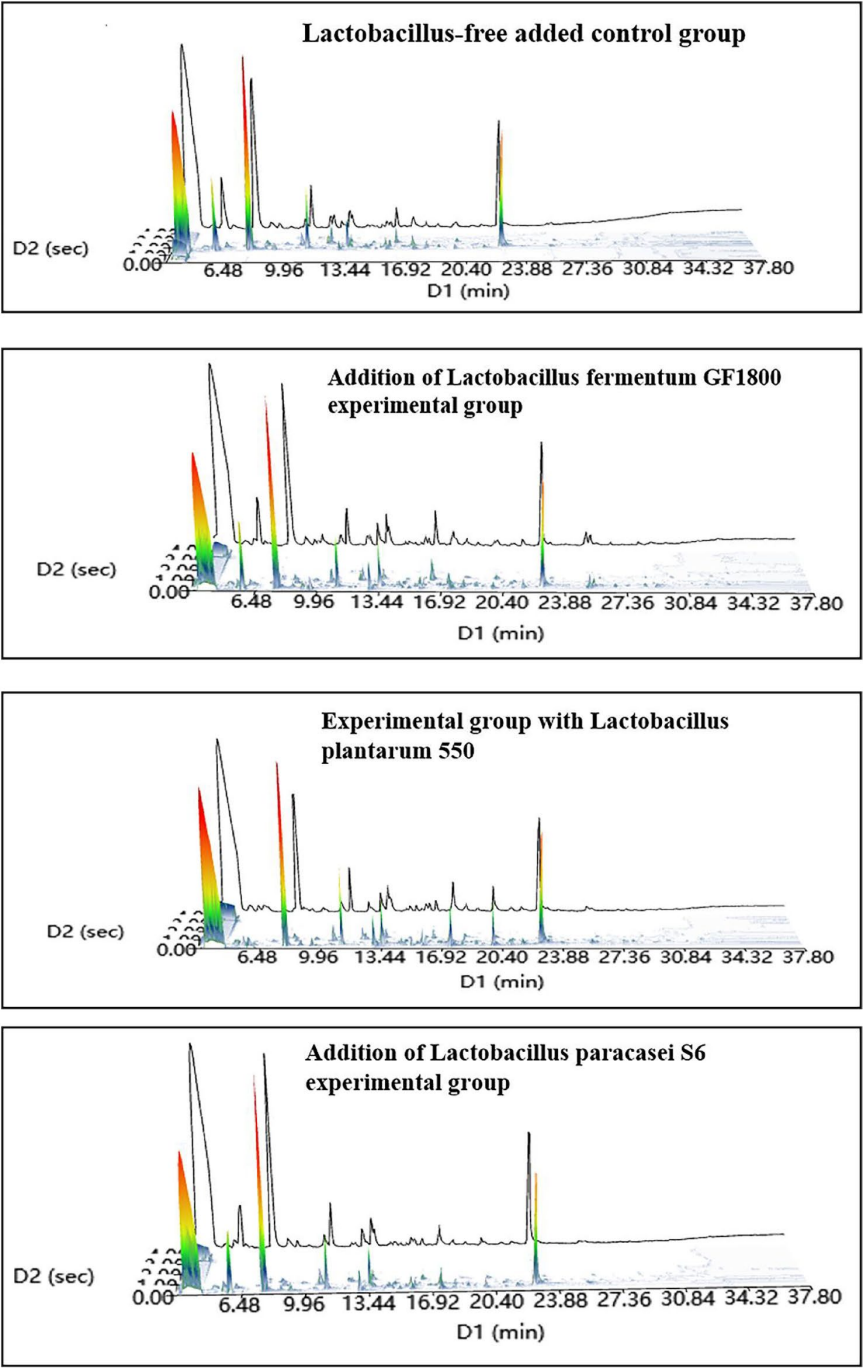
Comparison results of the relative content of various types of volatile substances in superfine indica rice samples fermented by different *Lactobacillus*.

Figure 3 presents the total ion chromatograms of volatile compounds in Danling Dongba indica rice slurry, prepared using ultrafine indica rice flour and fermented with different *Lactobacillus* strains. The results indicate that fermentation with these strains enhanced the diversity of volatile flavor compounds, while the retention times of major compounds remained consistent across all samples.

### 3.7 Analysis of Volatile Flavor Substances in Different *Lactobacillus* Indicus Fermented Indica Rice Slurrys

To assess the impact of *Lactobacillus* species on volatile profiles in Danling Dongba fermented *indica* rice slurry, volatile organic compounds (VOCs) were analyzed using solid‐phase microextraction comprehensive two‐dimensional gas chromatography–mass spectrometry (SPME‐GC×GC–MS). A total of 72 VOCs were identified across *Lactobacillus*‐fermented ultrafine *indica* rice slurries, categorized into seven classes: 6 hydrocarbons/alkenes and heterocyclic compounds, 24 alcohols, 9 aldehydes, 8 acids, 12 ketones, and 12 esters. In contrast, the control (no *Lactobacillus*) contained significantly fewer VOCs (42 compounds), with *L. paracasei* , *L. plantarum* , and *L. fermentum* yielding 51, 53, and 62 compounds respectively (Table 5). These results demonstrate that *Lactobacillus* fermentation significantly enriches VOC diversity in rice slurry.

**TABLE 5:** GC×GC–MS identification of volatile components in different *Lactobacillus* fermented ultra indica samples.

1 D retention time/min	2 D retention time/sec	Compound name	CAS No.	Relative peak area/%			
				FJ	FG	ZW	WR
Hydrocarbons, alkenes, heterocycles (6 types)							
14.48	0.76	Hexane, 1‐nitro‐	646‐14‐0	0.12 ± 0.02	0.06 ± 0.01	0 ± 0	0 ± 0
3.48	0.02	Methyl hydrogen disulfide	6251‐26‐9	0 ± 0	36.94 ± 0.71	0 ± 0	0 ± 0
3.94	0.68	Hydrazine, methyl‐	60–34‐4	1.24 ± 0.05	0.79 ± 0.08	0.27 ± 0.03	1.93 ± 0.03
15.94	0.59	Cyclopropane, pentyl‐	2511‐91‐3	0.56 ± 0.1	0.35 ± 0.03	0.61 ± 0.05	0.55 ± 0.04
37.39	2.22	1,4,7,10,13,16‐Hexaoxacyclooctadecane	17,455–13‐9	0 ± 0	0 ± 0	0 ± 0	0.38 ± 0.04
8.74	0.76	Styrene	100–42‐5	0.73 ± 0.11	0.46 ± 0.1	0.29 ± 0.01	0.52 ± 0.11
Alcohol (24 types)							
17.61	0.36	2‐Furanmethanol	98–00–0	0 ± 0	0.02 ± 0.01	0.12 ± 0.07	0 ± 0
5.74	0.14	1‐Propanol, 2‐methyl‐	78–83‐1	5.03 ± 0.22	3.39 ± 0.23	5.72 ± 0.32	5.32 ± 0.31
15.74	0.67	Linalool	78–70‐6	0.56 ± 0.07	0.34 ± 0.02	0.68 ± 0.06	0.49 ± 0.06
8.54	0.32	1‐Pentanol	71–41‐0	0.6 ± 0.08	0.35 ± 0.02	0.58 ± 0.04	0.75 ± 0.05
3.48	3.13	Ethanol	64–17‐5	25.28 ± 0.5	17.48 ± 0.43	27.1 ± 0.47	20.32 ± 0.89
4.08	0.28	Ethanol, 2‐nitro‐	625–48‐9	0 ± 0	0.23 ± 0.04	0.32 ± 0.09	0 ± 0
10.14	0.4	1‐Pentanol, 4‐methyl‐	626–89‐1	0 ± 0	0 ± 0	0.05 ± 0	0 ± 0
14.54	0.49	cis‐Hept‐4‐enol	6191‐71‐5	0.33 ± 0.04	0.21 ± 0	0.37 ± 0.04	0.3 ± 0.02
12.21	0.59	3‐Octanol	589–98‐0	0.06 ± 0	0.04 ± 0	0.07 ± 0.01	0 ± 0
20.88	0.73	(Z)‐4‐Decen‐1‐ol	57,074–37‐0	0.28 ± 0.03	0.17 ± 0.01	0.32 ± 0.03	0 ± 0
35.14	0.52	Heptaethylene glycol	5617‐32‐3	1.3 ± 0.13	0.1 ± 0.01	0.34 ± 0.04	8.47 ± 0.53
15.14	0.3	2,3‐Butanediol	513–85‐9	0.34 ± 0.05	0.29 ± 0.03	0.75 ± 0.04	0.31 ± 0.08
36.41	0.52	2‐[2‐[2‐[2‐[2‐[2‐[2‐(2‐Hydroxyethoxy)ethoxy] ethoxy]ethoxy]ethoxy]ethoxy]ethoxy] ethanol	5117–19‐1	0.35 ± 0.09	0 ± 0	0 ± 0	2.52 ± 0.09
18.88	0.51	1‐Propanol, 3‐(methylthio)‐	505–10‐2	0.23 ± 0.02	0.16 ± 0.01	0.26 ± 0.01	0.24 ± 0.03
13.41	0.52	1‐Octen‐3‐ol	3391‐86‐4	3 ± 0.08	2 ± 0.07	3.51 ± 0.24	2.37 ± 0.25
14.41	0.54	2‐Nonen‐1‐ol, (E)‐	31,502–14‐4	0.15 ± 0.03	0 ± 0	0 ± 0	0.14 ± 0.01
22.68	0.62	Phenylethyl Alcohol	1960‐12‐8	12.47 ± 0.9	8.17 ± 0.17	13.63 ± 0.39	12.9 ± 0.28
13.72	1.91	2‐Octen‐1‐ol, (E)‐	18,409–17‐1	0 ± 0	0 ± 0	0 ± 0	0.28 ± 0.04
18.19	2.05	1‐Nonanol		0.47 ± 0.06	0.29 ± 0.03	0.62 ± 0.06	0.52 ± 0.07
7.59	1.68	1‐Butanol, 3‐methyl‐		21.66 ± 0.33	13.14 ± 0.75	20.66 ± 0.49	21.71 ± 1.24
11.05	1.8	1‐Hexanol		3.65 ± 0.25	2.41 ± 0.06	4.08 ± 0.22	4.11 ± 0.2
14.41	0.55	1‐Hexanol, 2‐ethyl‐		0 ± 0	0 ± 0	0.15 ± 0.03	0 ± 0
18.59	2.02	3‐Nonen‐1‐ol, (Z)‐		0 ± 0	0 ± 0	0 ± 0	0.13 ± 0.01
20.99	2.1	3‐Decen‐1‐ol, (Z)‐		0 ± 0	0 ± 0	0 ± 0	0.23 ± 0.04
Aldehyde (9 types)							
5.41	0.31	Hexanal	66–25‐1	1.56 ± 0.07	0.97 ± 0.06	1.26 ± 0.12	1.24 ± 0.18
23.21	0.9	Benzeneacetaldehyde, α‐ethylidene‐	4411‐89‐6	0.17 ± 0.01	0.12 ± 0.01	0.2 ± 0.02	0 ± 0
13.14	0.89	2‐Octenal, (E)‐	2548‐87‐0	0 ± 0	0.06 ± 0.01	0.13 ± 0.04	0 ± 0
13.14	0.87	2‐Nonenal, (E)‐	18,829–56‐6	0.11 ± 0.04	0 ± 0	0.05 ± 0.01	0 ± 0
9.61	0.21	Acetaldehyde, hydroxy‐	141–46‐8	1.66 ± 0.09	0 ± 0	0.46 ± 0.04	0 ± 0
12.45	2.42	Nonanal	124–19‐6	0.97 ± 0.22	1.25 ± 0.17	1.61 ± 0.16	1.44 ± 0.2
17.32	2.11	Benzeneacetaldehyde	122–78‐1	1.36 ± 0.06	0.74 ± 0.02	1.65 ± 0.07	1.47 ± 0.19
14.99	2.49	Decanal	112–31‐2	0.08 ± 0	0.07 ± 0.02	0.18 ± 0.07	0.14 ± 0.03
14.81	0.69	Benzaldehyde	100–52‐7	0.22 ± 0.02	0.15 ± 0.02	0.29 ± 0.01	0.15 ± 0.03
Acids (8 types)							
15.74	0.28	Propanoic acid, 2‐methyl‐	79–31‐2	0.67 ± 0.08	0.54 ± 0.04	0.85 ± 0.11	0.6 ± 0.15
12.88	0.18	Acetic acid	64–19‐7	2.2 ± 0.17	1.72 ± 0.19	3.39 ± 0.21	1.56 ± 0.25
13.01	0.36	Butanedioic acid, 2,3‐bis(acetyloxy)‐, [R‐(R*,R*)]‐	51,591–38‐9	0.1 ± 0.04	0 ± 0	0.03 ± 0.02	0 ± 0
29.34	0.64	n‐Decanoic acid	334–48‐5	0 ± 0	0 ± 0	0.22 ± 0.02	0 ± 0
21.59	1.78	Hexanoic acid	142–62‐1	0.7 ± 0.16	0.44 ± 0.03	1.01 ± 0.08	0.58 ± 0.07
25.65	1.9	Octanoic acid	124–07‐2	1.47 ± 0.15	0 ± 0	1.16 ± 0.08	0.5 ± 0.04
21.08	0.94	Acetic acid, 2‐phenylethyl ester	103–45‐7	0.22 ± 0.04	0.15 ± 0.01	0.26 ± 0.02	0.21 ± 0.01
21.94	0.52	N‐carbobenzyloxy‐l‐tyrosyl‐l‐valine	0–0‐0	0 ± 0	0.02 ± 0	0.03 ± 0	0 ± 0
Ketone (12 types)							
25.34	0.42	Dihydroxyacetone	96–26‐4	1.54 ± 0.09	0 ± 0	0 ± 0	0 ± 0
10.68	0.85	6‐Octen‐2‐one, (Z)‐	74,810–53‐0	1.57 ± 0.11	1.16 ± 0.12	1.64 ± 0.06	1.26 ± 0.19
5.01	0.19	2,3‐Pentanedione	600–14‐6	0.09 ± 0.01	0.09 ± 0.01	0.13 ± 0.01	0.1 ± 0.01
9.08	0.28	Acetoin	513–86‐0	0 ± 0	0.41 ± 0.05	0 ± 0	0 ± 0
16.21	0.78	3,5‐Octadien‐2‐one	38,284–27‐4	0.12 ± 0.01	0.05 ± 0	0.09 ± 0	0.09 ± 0.03
22.48	1.13	5,9‐Undecadien‐2‐one, 6,10‐dimethyl‐, (E)‐	3796‐70‐1	0.06 ± 0.01	0 ± 0	0 ± 0	0 ± 0
28.48	0.58	4H‐Pyran‐4‐one, 2,3‐dihydro‐3,5‐dihydroxy‐6‐methyl‐	28,564–83‐2	0.28 ± 0.05	0 ± 0	0 ± 0	0 ± 0
19.81	0.48	1,2‐Cyclopentanedione	3008–40–0	0 ± 0	0 ± 0	0.06 ± 0.01	0 ± 0
14.85	2.15	3 (2H)‐Thiophenone, dihydro‐2‐methyl‐	13,679–85‐1	0.15 ± 0.01	0.13 ± 0.01	0.19 ± 0.02	0.15 ± 0.01
9.74	0.93	2‐Octanone	111–13‐7	0 ± 0	0.29 ± 0.06	0 ± 0	0 ± 0
11.05	2.16	5‐Hepten‐2‐one, 6‐methyl‐	110–93‐0	0 ± 0	0 ± 0	0 ± 0	0.25 ± 0
7.32	2.13	2‐Hexanone, 5‐methyl‐	110–12‐3	0.25 ± 0.05	0.14 ± 0.03	0.19 ± 0.06	0.28 ± 0.02
Esters (12 types)							
26.81	0.51	2‐Hydroxy‐gamma‐butyrolactone	19,444–84‐9	0.44 ± 0.14	0 ± 0	0 ± 0	0 ± 0
3.05	1.54	Ethyl acetate	141–78‐6	1.43 ± 0.1	1.06 ± 0.09	0 ± 0	1.51 ± 0.09
6.05	1.93	1‐Butanol, 3‐methyl‐, acetate	123–92‐2	0.81 ± 0.14	0.59 ± 0.11	0.56 ± 0.03	0.84 ± 0.12
8.59	2.45	Hexanoic acid, ethyl ester	123–66‐0	0.75 ± 0.14	0.46 ± 0.08	0.6 ± 0.04	0.59 ± 0.05
15.99	2.65	Nonanoic acid, ethyl ester	123–29‐5	0.08 ± 0.01	0.06 ± 0.01	0.12 ± 0.02	0.11 ± 0.02
18.59	2.64	Decanoic acid, ethyl ester	110–38‐3	0.36 ± 0.07	0.18 ± 0.02	0.45 ± 0.06	0.29 ± 0.04
4.54	0.39	Isobutyl acetate	110–19‐0	0 ± 0	0 ± 0	0.12 ± 0.01	0 ± 0
13.61	1.24	Octanoic acid, ethyl ester	106–32‐1	1.18 ± 0.14	0.67 ± 0.04	1.08 ± 0.09	0.85 ± 0.08
11.08	1.16	Heptanoic acid, ethyl ester	106–30‐9	0.12 ± 0.01	0.07 ± 0.02	0.09 ± 0.03	0 ± 0
25.21	0.94	2 (3H)‐Furanone, dihydro‐5‐pentyl‐	104–61‐0	0.1 ± 0.02	0 ± 0	0.07 ± 0.01	0 ± 0
20.48	0.97	Benzeneacetic acid, ethyl ester	101–97‐3	0.06 ± 0.02	0.02 ± 0	0.07 ± 0.01	0 ± 0
19.88	0.33	Oxime‐, methoxy‐phenyl‐	0–0‐0	0.73 ± 0.16	1 ± 0.07	1.2 ± 0.14	1.28 ± 0.28
Phenolic (1 types)							
29.6087	0.854	2,4‐Di‐tert‐butylphenol	96–76‐4	0 ± 0	0.02 ± 0	0.03 ± 0.01	0 ± 0

**FIGURE 4:**
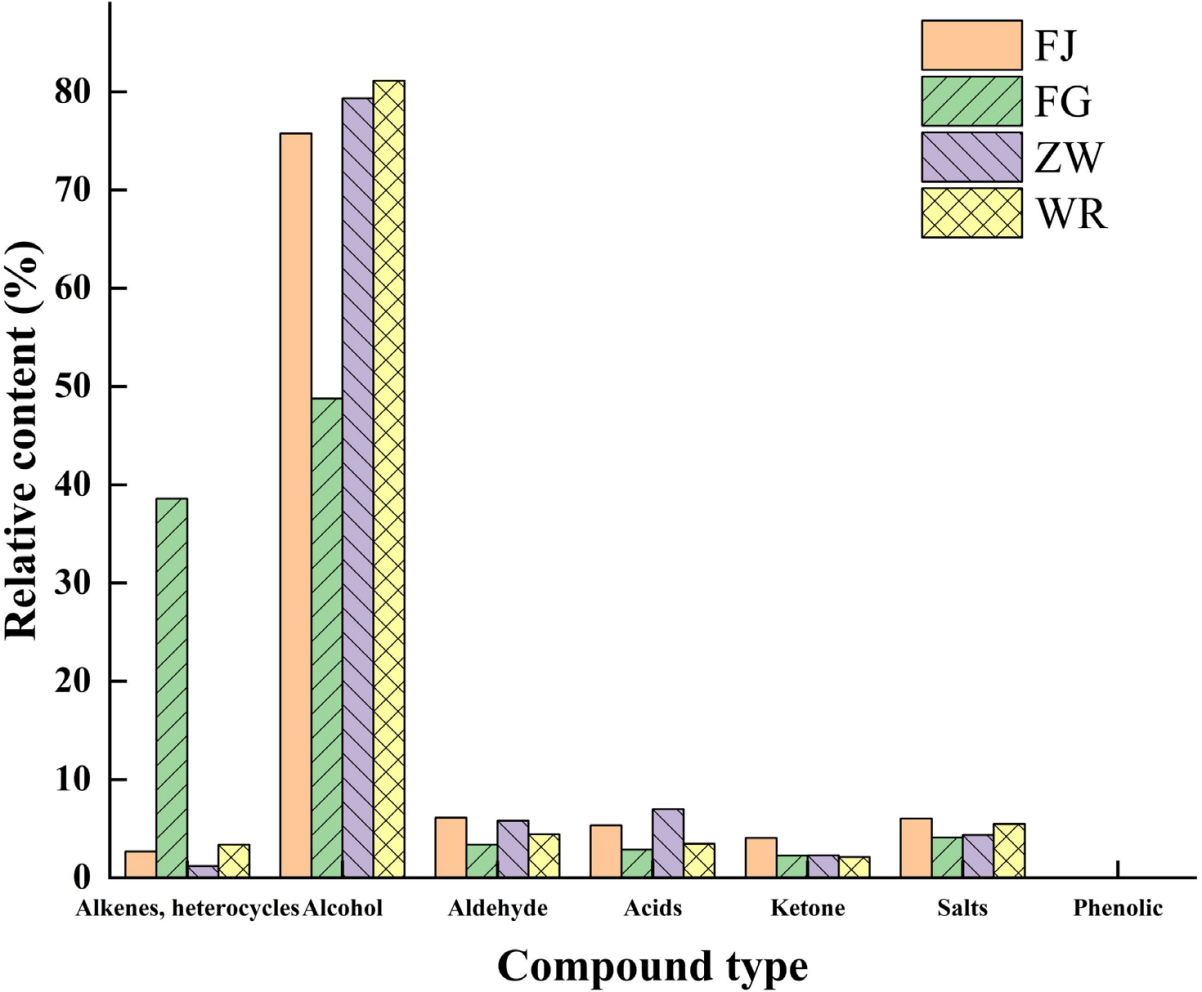
Comparison results of the relative content of various types of volatile substances in superfine indica rice samples fermented by different *Lactobacillus*.

Ester biosynthesis primarily occurs via alcohol–acid esterification reactions. These key flavor compounds impart characteristic sweet and fruity aromas to fermented products (Xu et al. 2022). As evidenced in Tables 5 and 6, *Lactobacillus*‐fermented samples exhibited significantly greater diversity and higher relative abundance of volatile compounds compared to yeast‐fermented controls. Among all volatile classes, alcohols demonstrated the highest mean relative abundance, dominated by ethanol, isoamyl alcohol, 2‐phenylethanol, isobutanol, n‐hexanol, and 1‐vinylhexanol. Notably, *L. paracasei* ‐fermented samples showed substantially elevated relative abundance of alkenes and heterocyclic compounds (38.59% of total volatiles) versus other treatments. Within this fraction, disulfanylmethane—detected exclusively in this group—accounted for 36.94% ± 0.71% of total volatiles. Furthermore, *L. fermentum* fermentation generated both greater diversity and higher relative abundance of aldehydes, acids, ketones, and esters (Figure 4), demonstrating strain‐specific modulation of flavor profiles during rice slurry fermentation.

**TABLE 6:** Distribution of various volatile components in supermicro *indica* rice samples fermented by different *Lactobacillus*.

Ingredient category	Number of classifications/kinds (average relative content/%)			
	FJ	FG	ZW	WR
Hydrocarbon‐alkene heterocycles	4 (2.66)	5 (38.59)	3 (1.18)	4 (3.38)
Alcohol	17 (75.76)	17 (48.79)	19 (79.34)	18 (81.13)
Aldehyde	8 (6.13)	7 (3.36)	9 (5.82)	5 (4.44)
Acids	6 (5.34)	5 (2.86)	8 (6.97)	6 (3.45)
Ketone	8 (4.06)	7 (2.26)	6 (2.30)	6 (2.12)
Esters	11 (6.05)	9 (4.11)	10 (4.36)	7 (5.48)
Phenolic	0 (0.00)	1 (0.02)	1 (0.03)	0 (0.00)
Total (grand)	62	51	53	42

**FIGURE 5:**
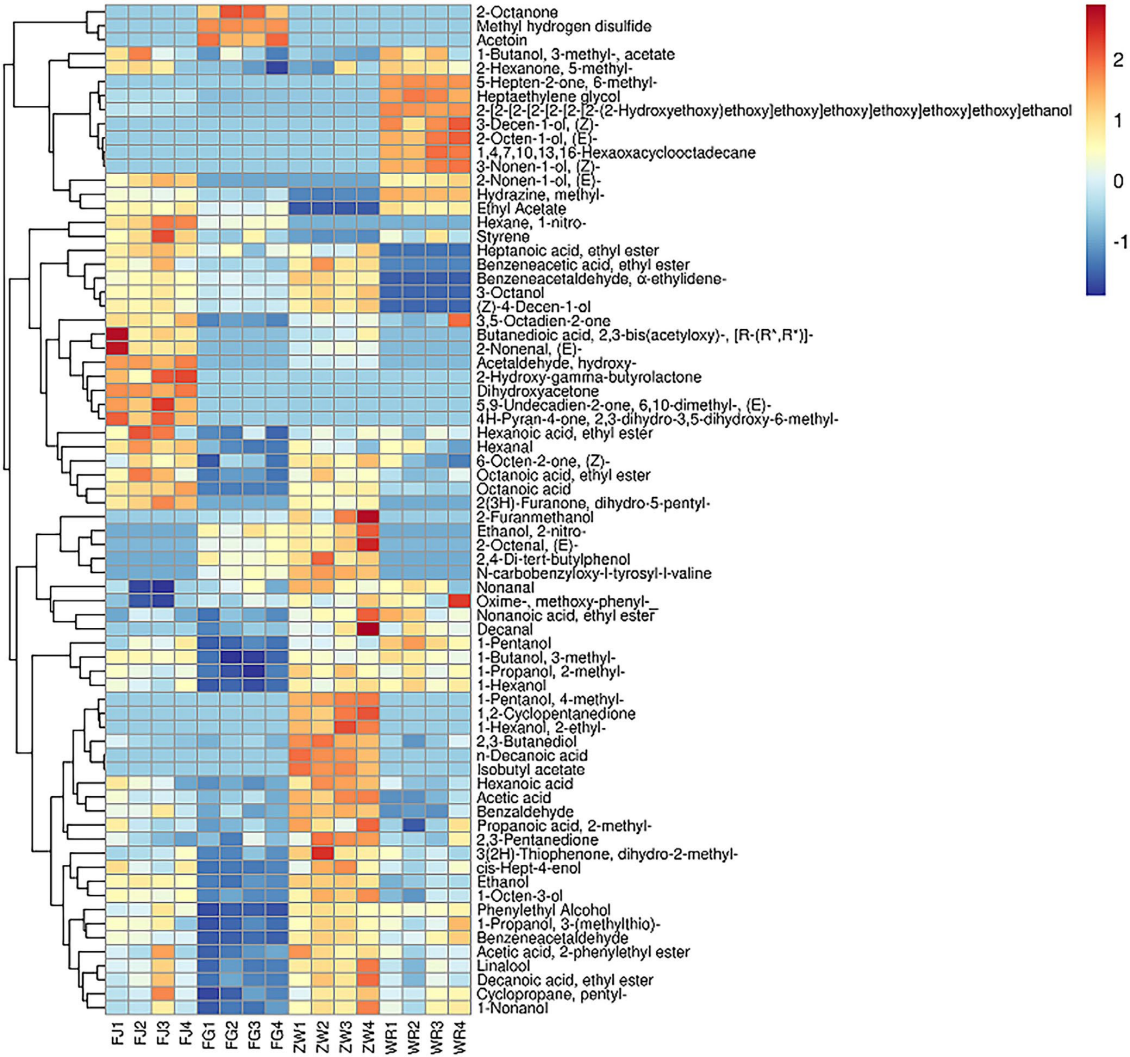
Heat map of volatile components of supermicro indica rice samples fermented with and without *Lactobacillus*. Red and blue colors represent above and below average metabolites, respectively; the redder the color, the higher the level.

*Lactobacillus paracasei* catalyzes the conversion of pyruvate to acetoin via enzymatic reactions, which is subsequently transformed into 3‐hydroxy‐2‐butanone. This compound imparts a creamy aroma typical of dairy products and significantly enhances the milk‐like flavor profile of fermented rice slurry. Additionally, *L. paracasei* generates flavor‐active compounds such as disulfanylmethane through sugar metabolism, which confers distinctive woody and yogurt‐like notes to the fermented product. As shown in Figure 5, cluster analysis of volatile compounds in *L. paracasei* ‐fermented ultrafine indica rice slurry revealed elevated concentrations of 3‐hydroxy‐2‐butanone (exhibiting butterscotch, woody, and yogurt‐like aromas), 2‐octanone (fatty aroma), and disulfanylmethane relative to other treatments. These findings indicate that *L. paracasei* plays a key role in the production of ketones (notably 3‐hydroxy‐2‐butanone) during rice slurry fermentation. Such metabolites enhance the sensory quality of rice‐based probiotic functional foods, supporting their potential as viable dairy alternatives (Moiseenko et al. 2024).

*Lactobacillus plantarum* generates ester and aldehyde volatiles via its esterases and acidifying enzymes. Specifically, oxidation of ethanol and lactic acid to acetic acid or acetaldehyde enhances ester aroma formation while increasing slurry acidity and flavor complexity. In *L. plantarum* ‐fermented samples, significant increases in ethyl isobutyrate and ethyl caprylate concentrations conferred fruity, floral, vanilla, and apple aromas. Studies demonstrate that *L. plantarum's* esterases produce diverse fruit‐ and floral‐scented volatile alcohols during fermentation, which critically enhance fermented product sensory properties (Ghosh et al. 2021).

*Lactobacillus fermentum* generates diverse volatile compounds via its esterases, aldehyde dehydrogenases, and ketone‐forming enzymes. Key metabolites include α‐hydroxy‐γ‐butyrolactone, ethyl caprylate (pineapple, pear, floral), ethyl heptanoate (fruity, wine‐like), γ‐nonolactone, phenylethyl acetate (floral, fruity, honey, rose), and 1,3‐dihydroxyacetone (mint, cooling). These metabolic products significantly elevate ester, aldehyde, and ketone concentrations in fermented samples, thereby enhancing flavor complexity. Studies confirm that such esters constitute critical volatile flavor compounds in fermented foods, imparting sweet, fruity sensory attributes (van Wyk 2024).

**FIGURE 6:**
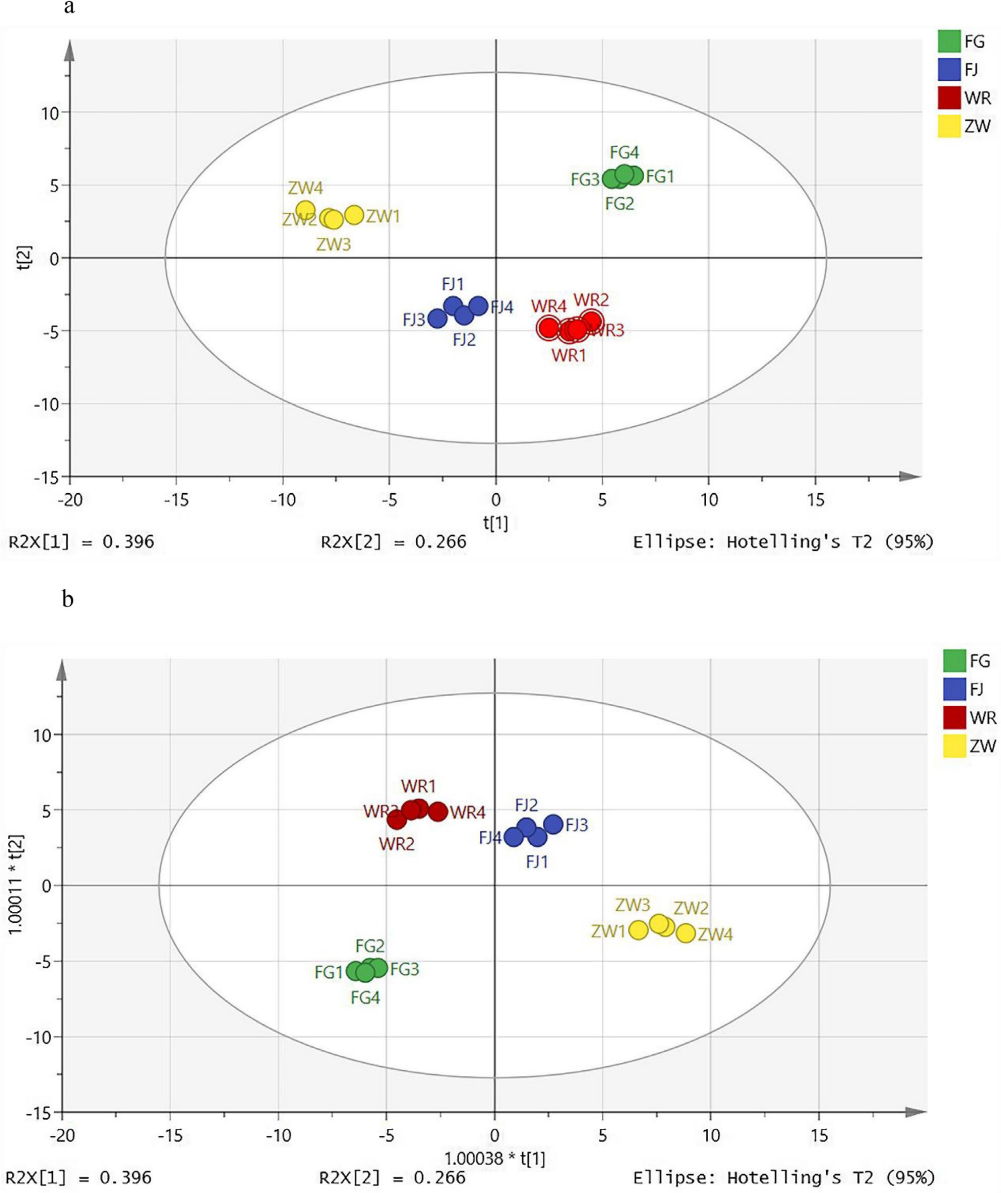
Principal component analysis graph of *indica* rice samples fermented with different *Lactobacillus* (a). OPLS‐DA diagram of *indica* rice samples fermented with different *Lactobacillus* (b). “FJ” denotes fermented *Lactobacillus paracasei* GF1800, “FG” denotes *Lactobacillus paracasei* S8, “ZW” denotes *Lactobacillus plantarum* . “WR” means without *Lactobacillus fermentum* .

Principal Component Analysis (PCA) is a dimensionality reduction technique that compresses datasets while preserving essential information. It effectively highlights similarities and differences among datasets, often visualized through score plots (Kong et al. 2025). Figure 6a presents the PCA results for the indica rice samples spiked with different *Lactobacillus* species. The three principal components in the model accounted for a total of 88.3% of the variance, with contributions of 39.6% for PC1, 26.6% for PC2, and 22.1% for PC3. These results suggest that the model effectively captured the majority of the information regarding the volatile components in the samples. The correlation score plots revealed that the four samples were distributed across different quadrants, indicating that the various *Lactobacillus* species exerted distinct effects on the volatile profiles of the fermented indica samples, thereby explaining the differences observed among the treatment groups.

**FIGURE 7:**
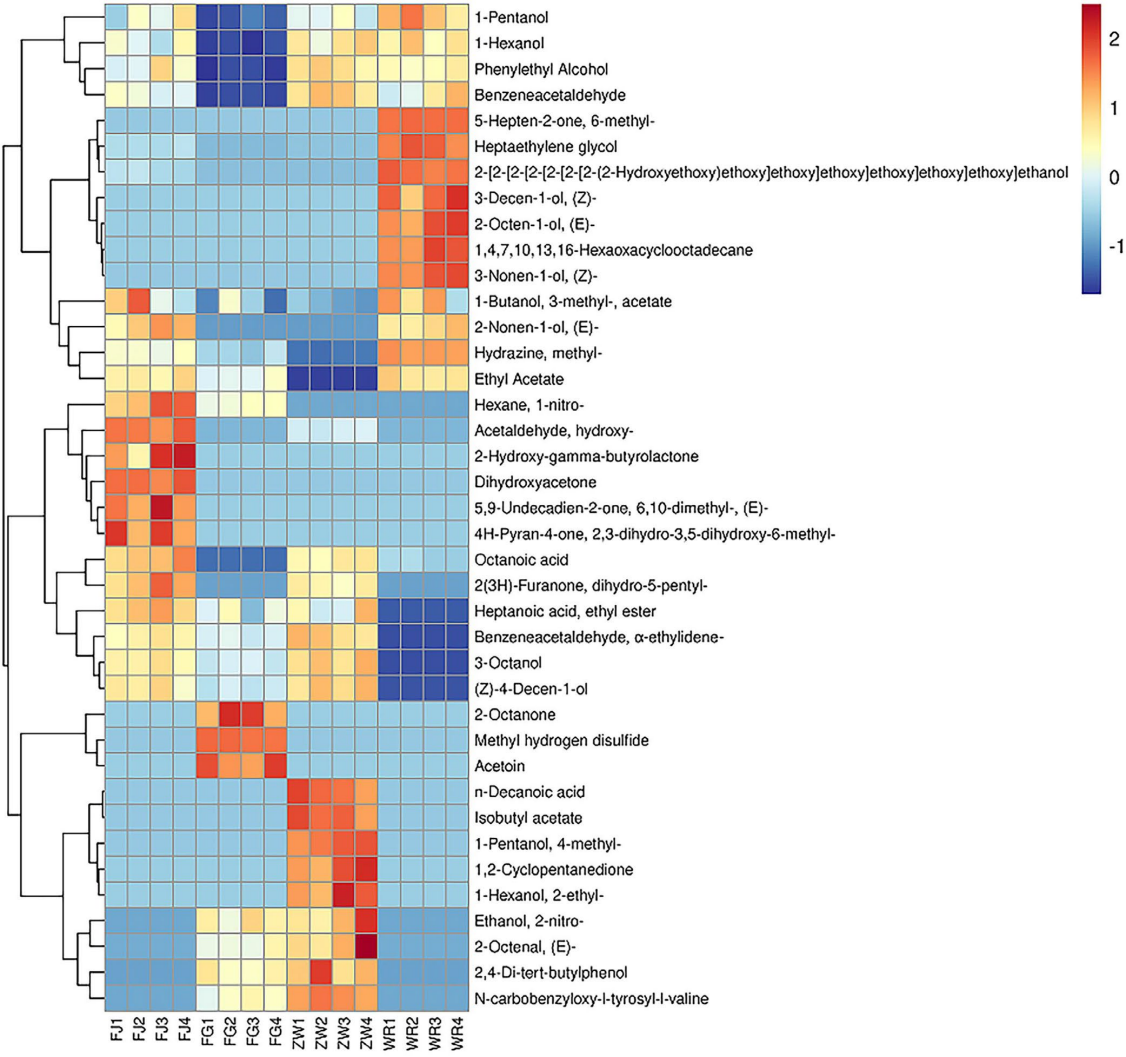
ISDM clustering heat map of fermented supermicro *Indica* rice samples added with.

The metabolic fingerprints of the groups with and without the addition of *Lactobacillus* were compared using an Orthogonal Partial Least Squares Discriminant Analysis (OPLS‐DA) model. The model exhibited *R* ^2^ *Y* and *Q* ^2^ values of 0.883 and 0.983, respectively, both close to 1, indicating a strong model fit. As shown in Figure 6b, the addition of *Lactobacillus* significantly influenced the volatile flavor profiles of fermented indica rice, with the groups distinctly distributed across different quadrants. Through OPLS‐DA analysis, 39 volatiles were identified as key indicators of differences between the groups, including 1‐nitrohexane (VIP = 1.13), geranylacetone (VIP = 1.13), and 6‐methyl‐5‐hepten‐2‐one (VIP = 1.11). Based on VIP values (> 1) and *p*‐values (< 0.05), 36 significantly different metabolites (ISDMs) were identified. These ISDMs are visually represented in a clustered heat map (Figure 7).

The results indicated that the control group, which lacked *Lactobacillus*, exhibited a higher relative content of volatile metabolites, particularly alcohols such as (Z)‐3‐decenol and (E)‐2‐octanol. These compounds are known for their pungent aroma, which may negatively impact the flavor profile of Danling Dongba (Zhao et al. 2020).

The samples spiked with *Lactobacillus paracasei* exhibited high relative contents of volatile metabolites, including 3‐hydroxy‐2‐butanone, 2‐octanone, and disulfanylmethane. These ketones are primarily associated with sweet, creamy, or buttery flavors, suggesting that *Lactobacillus paracasei* hydrolyzes starch sugars through its enzymatic activity, leading to the formation of volatile compounds characteristic of Danling Dongba (Guo et al. 2020). In the samples spiked with *Lactobacillus plantarum* , the relative contents of isobutyl acetate (characterized by apple, banana, floral, and vanilla aromas), 1,2‐cyclopentanedione, and caprylic acid were significantly higher. These esters, ketones, and aldehydes are critical contributors to the flavor profile of tannin frozen poi, significantly influencing the overall flavor due to their low sensory threshold values. *Lactobacillus plantarum* facilitates the production of isobutyl acetate through the release of esterases, thereby enhancing the intensity of volatile compounds (Wang et al. 2020).

Additionally, samples spiked with *Lactobacillus fermentum* exhibited higher relative levels of volatiles such as α‐hydroxy‐γ‐butyrolactone, 1,3‐dihydroxyaceto‐ne, and geranylacetone. Analysis revealed that 18 significantly different metabolites (ISDMs) were identified in samples fermented with yeast alone, while 20, 19, and 23 ISDMs were detected in samples spiked with *L. paracasei* GF1800, *L. fermentum* S6, and *L. plantarum 550*, respectively. Furthermore, it was noted that the primary microbial constituents of the fermented indica rice slurry in Danling Dongba are lactic acid bacteria (LAB) and yeast. These microorganisms are responsible for producing most of the organic acids and alcoholic compounds while contributing to aroma formation through the synthesis of minor acids and aldehydes or the action of their hydrolytic enzymes (Liu et al. 2020).

## 4 Conclusion

In summary, the ultrafine indica rice flour with an average particle size of 12.22 μm demonstrated superior water and oil absorption capacities, solubility, and pasting viscosity, indicating its potential to improve texture during fermentation. Additionally, fermentation with *Lactobacillus* significantly enhanced the volatile flavor complexity of rice slurry, increasing the number and relative content of key aroma compounds such as esters, alcohols, and sulfur‐containing substances. In particular, *L. plantarum, L. fermentum*, and *L. paracasei* contributed distinctively to flavor enhancement. Multivariate analyses (PCA and OPLS‐DA) confirmed clear separations in volatile metabolite profiles between treated and control groups. These findings support the optimization of flour fineness and strain selection to enhance Danling Dongba's flavor and processing qualities, facilitating its industrial application and consumer appeal.

## Author Contributions

**Hong Jiang:** investigation (equal), methodology (equal), writing – original draft (equal), writing – review and editing (equal). **Chong Liu:** investigation (equal), methodology (equal), writing – original draft (equal), writing – review and editing (equal). **Hailun Wan:** formal analysis (equal), investigation (equal), methodology (equal). **Hongyu Li:** conceptualization (equal), supervision (equal). **Zhangkui Yan:** conceptualization (equal), supervision (equal). **Xiaohong Wen:** formal analysis (equal), investigation (equal), methodology (equal). **Wanqing Feng:** conceptualization (equal), investigation (equal), methodology (equal). **Can Tang:** formal analysis (equal), investigation (equal), methodology (equal). **Yong Tang:** conceptualization (equal), funding acquisition (equal), supervision (equal), writing – review and editing (equal).

## Ethics Statement

The authors have nothing to report.

## Conflicts of Interest

The authors declare no conflicts of interest.

## Data Availability

The datasets used and/or analyzed in this study are available from the corresponding author upon reasonable request.
